# Tumor Infiltrating Lymphocytes in Cutaneous Squamous Cell Carcinoma—A Systematic Review

**DOI:** 10.3390/dermatopathology13010006

**Published:** 2026-01-13

**Authors:** Li Yang Loo, Shi Huan Tay, Choon Chiat Oh

**Affiliations:** 1 Department of Dermatology, Singapore General Hospital, Singapore 169608, Singapore; 2 SingHealth Duke-NUS Academic Medical Centre, Singapore 169856, Singapore

**Keywords:** squamous cell carcinoma, tumor-infiltrating lymphocytes, immunosuppression, tumor microenvironment, immunology

## Abstract

Cutaneous squamous cell carcinoma (cSCC) is an immunogenic malignancy with variable immune infiltration and inconsistent responses to checkpoint blockade. Tumor-infiltrating lymphocytes (TILs) influence tumor progression and therapeutic outcome, yet their phenotypic and functional diversity across disease contexts remains incompletely understood. This review systematically characterizes the TIL landscape in human cSCC. Following PRISMA 2020 guidelines, PubMed and Embase were searched up to May 2025 and restricted to studies evaluating tumor-infiltrating lymphocytes in human cSCC, using the modified Newcatle–Ottawa score to assess risk of bias. Data were synthesized qualitatively given methodological heterogeneity. 48 studies met inclusion criteria. cSCCs exhibited dense CD3^+^ infiltrates composed of cytotoxic (CD8^+^GzmB^+^, Ki-67^+^, CD69^+^) and regulatory (FOXP3^+^, CCR4^+^) subsets. Higher CD8^+^ activity correlated with smaller tumors and longer disease-free survival, whereas FOXP3^+^ enrichment and TGF-β2 signaling promoted immune evasion. Immunosuppressed patients demonstrated diminished CD8^+^ density and clonality. Immune modulation with PD-1/PD-L1 blockade, imiquimod, HPV vaccination, or OX40 stimulation enhanced effector function. The cSCC immune microenvironment reflects a balance between cytotoxic and suppressive factors. Harmonizing multimodal immune profiling and integrating spatial context with systemic immune status may advance both prognostic stratification and therapeutic design.

## Simple Summary

Squamous cell carcinoma is a common skin cancer in which the body’s immune system plays an important role in controlling tumor growth. This cancer contains large numbers of immune cells called tumor-infiltrating lymphocytes. Theoretically, these immune cells are supposed to eliminate cancer, but they do not always work effectively. In this review, we examined decades of research to understand which immune cells help restrain cancer and which allow the cancer to progress. Killer T-cells, a type of tumor-infiltrating lymphocyte, can limit tumor growth when they are active and able to reach tumor cells. Regulatory and suppressive T-cells, along with tumor-associated signals, can block this response. Importantly, immune “brakes” targeted by modern immunotherapies often mark active immune responses rather than immune failure. These findings help clarify why immunotherapy works in some patients but not others and highlight new immune targets. This knowledge supports improved diagnostic interpretation and guides future research toward more precise, personalized immune-based treatments in squamous cell carcinoma.

## 1. Introduction

Cutaneous squamous cell carcinoma (cSCC) is the second most common non-melanoma skin cancer with rising incidence worldwide [1].

Cutaneous squamous cell carcinoma (cSCC) represents one of the most immunogenic forms of nonmelanoma skin cancer, arising in a setting where ultraviolet radiation, chronic inflammation, viral infection, and immunosuppression collectively drive malignant transformation of keratinocytes [2]. The tumor microenvironment (TME) of cSCC comprises a complex and dynamic interplay between tumor-infiltrating lymphocytes (TILs), stromal elements, and immune checkpoints that collectively determine whether the immune response suppresses or facilitates tumor progression [3]. This intricate balance between immune activation and immune evasion ultimately determines clinical outcomes ranging from local control to aggressive, metastatic disease [4].

Over the past three decades, histological, immunohistochemical, and, more recently, single-cell transcriptomic studies have revealed remarkable heterogeneity in the immune infiltrate of cSCC. Tumor-infiltrating lymphocytes (TILs), particularly CD8^+^ cytotoxic T-cells and CD4^+^ helper subsets, are central to tumor immunosurveillance and correlate with improved outcomes. Conversely, increased infiltration by FOXP3^+^ regulatory T-cells (Tregs), expression of immune checkpoints such as PD-1, PD-L1, LAG-3, and VISTA, and the presence of immunosuppressive cytokine networks such as TGF-β2 contribute to immune evasion and resistance to therapy. The balance between these factors defines the immune equilibrium of cSCC, which can shift toward either tumor restraint or tumor permissiveness depending on local and systemic factors.

Immunosuppressed populations, including solid organ transplant recipients and those receiving chronic immunosuppressive therapy, demonstrate a higher incidence, multiplicity, and aggressiveness of cSCC [5]. These groups exhibit an altered TME characterized by diminished effector T-cell infiltration, reduced cytotoxic activity, and expansion of suppressive immune subsets. Despite their high disease burden, solid organ transplant recipients have been excluded from many clinical trials involving immune checkpoint inhibitors (ICIs) due to concerns regarding their limited efficacy and the substantial risk of precipitating allograft rejection [6]. Case series have indicated that allograft rejection can occur even after administration of PD-1 or CTLA-4 blockade [7]. Consequently, this exclusion has created a critical knowledge gap in understanding how systemic immunosuppression reshapes cSCC immunity and influences responsiveness to emerging immunotherapies.

At present, T-cell–directed therapies form the cornerstone of immunomodulatory approaches in advanced cSCC [8]. Immune checkpoint inhibitors targeting PD-1 (e.g., cemiplimab and pembrolizumab) have shown efficacy in metastatic or unresectable disease, producing durable responses that highlight the fundamental role of cytotoxic T lymphocytes in cSCC control. Building on this success, adoptive T-cell therapies, including ex vivo expansion of tumor-reactive TILs and engineered T-cell receptor (TCR) or chimeric antigen receptor (CAR) platforms, are being trialed in melanoma [9]. These strategies aim to restore or augment tumor-specific T-cell activity within an otherwise immunosuppressive TME. Early translational studies have identified p53-and HPV-specific T-cell clones capable of recognizing cSCC tumor antigens, supporting the feasibility of precision T-cell-based interventions [10].

TILs reflect the host immune response to tumor antigens, encompassing diverse lymphocyte subsets with varying functional phenotypes—from cytotoxic effector to regulatory and exhausted states. In cSCC, characterizing TIL density, distribution, and subtype composition offers insights into disease biology, prognosis, and potential immunotherapeutic targets.

This review synthesizes existing evidence on the density and immunophenotypic characteristics of TILs in human cutaneous SCC, delineating the cellular and molecular features of the cSCC immune landscape. We further examine tumor-restraining and tumor-permissive lymphocyte subsets, the influence of systemic immune competence, and the effects of external immunomodulatory factors, before proposing an integrated framework for future immunopathologic and therapeutic research.

## 2. Materials and Methods

This systematic review was conducted according to the Preferred Reporting Items for Systematic Reviews and Meta-Analyses (PRISMA) guidelines for systematic reviews [11] (PRISMA 2020 for Abstracts Checklist in Supplementary Materials). This review was registered on the International Platform of Registered Systematic Review and Meta-analysis Protocols (INPLASY, ID: INPLASY202590114, 2025-9-27). A comprehensive literature search was conducted in PubMed and Embase using the search terms: (“tumor infiltrating lymphocytes” [All Fields] OR “lymphocytes, tumor infiltrating” [MeSH Terms] OR (“lymphocytes” [All Fields] AND “tumor infiltrating” [All Fields]) OR “tumor-infiltrating lymphocytes” [All Fields] OR (“tumor” [All Fields] AND “infiltrating” [All Fields] AND “lymphocytes” [All Fields]) OR “tumor infiltrating lymphocytes” [All Fields]) AND (“cutaneous” [All Fields] OR “cutaneously” [All Fields] OR “cutanous” [All Fields]) AND (“neoplasms, squamous cell” [MeSH Terms] OR (“neoplasms”[All Fields] AND “squamous” [All Fields] AND “cell” [All Fields]) OR “squamous cell neoplasms” [All Fields] OR (“squamous” [All Fields] AND “cell” [All Fields] AND “cancer” [All Fields]) OR “squamous cell cancer” [All Fields] OR “carcinoma, squamous cell” [MeSH Terms] OR (“carcinoma” [All Fields] AND “squamous” [All Fields] AND “cell” [All Fields]) OR “squamous cell carcinoma” [All Fields]). Studies in all languages after 1980 were included. We included studies that had histologically confirmed cSCC, were based on human tissue (primary or metastatic), and had quantitative or qualitative assessment of TILs or lymphocyte subsets (by immunohistochemistry (IHC), immunofluorescence, or flow cytometry). Studies were excluded if they were non-human or in vitro studies, focused on non-cutaneous SCCs (e.g., mucosal, head and neck), or were review articles, case reports without immunophenotyping, or papers limited to cell lines.

All identified publications were transferred to Rayyan [12], a systematic review management platform, and duplicates were removed manually. Google Translate was used to translate papers from other languages into English. Two reviewers (LLY, TSH) independently screened titles and abstracts based on the criteria described below, with full texts retrieved for articles deemed relevant. Full texts were screened again according to eligibility criteria. The quality of included publications was assessed by two authors using the modified Newcastle–Ottawa scale, with a score of four to be the minimum for selection [13], according to previously utilized methodology [14]. Any discrepancies were resolved through a third reviewer (OCC). Data extracted included total TIL density, distribution (intratumoral, peritumoral), expression of specific markers defining T-cell subsets (e.g., CD3, CD4, CD8, FOXP3, CD103, PD-1, and PD-L1), and characteristics of TILs modulated by therapeutic agents in cSCC. Where numerical data were not explicitly reported, results were synthesized qualitatively based on directionality and relative enrichment. No numerical values were estimated from graphical figures. Reported immune cell metrics were presented using the units provided by the original studies, without conversion, due to methodological heterogeneity. Effect sizes were reported using the metrics provided by individual studies (e.g., percentage of positive cells, fold change, or cell density), without conversion across units due to methodological heterogeneity. Given the heterogeneity of reporting, a narrative synthesis approach was employed. Quantitative comparison (meta-analysis) was not feasible due to methodological variability.

## 3. Results

**Figure 1:**
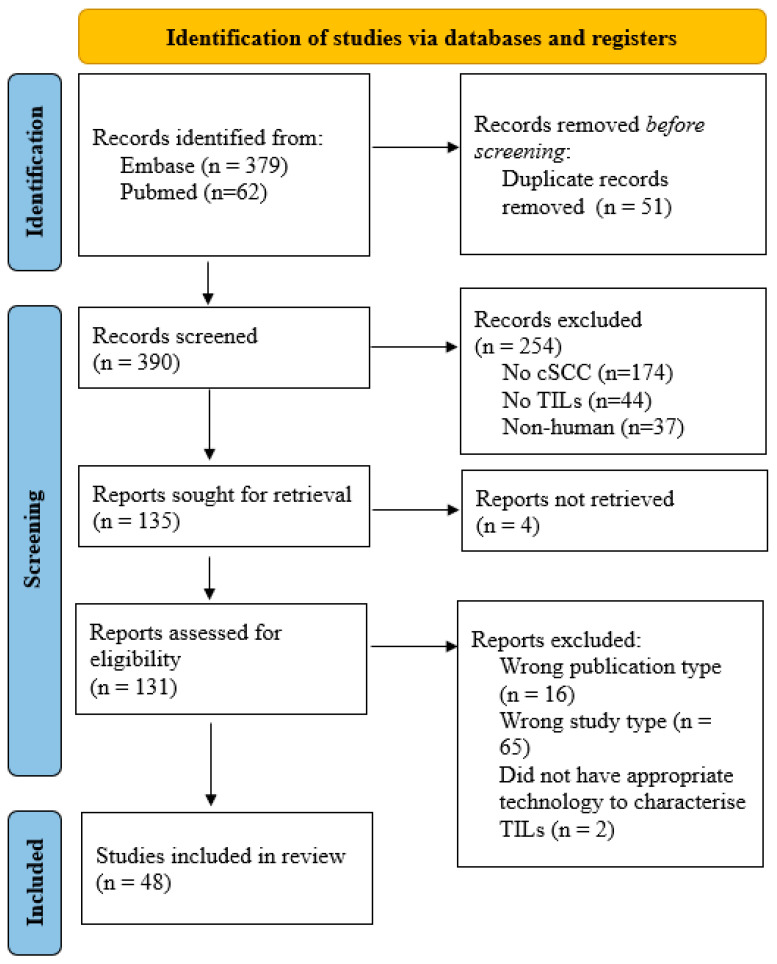
PRISMA Flow Diagram. The wrong publication type included abstracts and protocols. The wrong study type included review articles.

Out of 441 studies found from Embase (379) and Pubmed (62), 51 were duplicates. Of the remaining 390, 254 were excluded as the studies were on the wrong population (no cSCC: 174, no TILs: 44, nonhuman: 37). Of the remaining 135, 4 could not be obtained. 16 were the wrong publication type (14 abstracts and two study protocols) and thus excluded, while 65 were excluded as they were review articles. Of the remaining 54, six were excluded for the listed reasons (four could not be obtained, two were excluded as they did not characterize TILs). 48 studies were included, encompassing both primary and metastatic cutaneous squamous cell carcinoma (cSCC) samples, with comparisons to adjacent or normal skin where available. These results are summarized in the PRISMA Flow Diagram in Figure 1.

Sample sizes ranged from small translational studies to larger immunohistochemical series. Most studies were conducted in immunocompetent patients, though several included immunosuppressed cohorts, such as kidney transplant recipients. Data were synthesized qualitatively due to heterogeneous study designs, techniques, and outcome measures.

### 3.1. Characteristics of Tumor-Infiltrating Lymphocytes in Cutaneous Squamous Cell Carcinoma

Across the 48 included studies, tumor-infiltrating lymphocytes (TILs) in cSCC demonstrated substantial inter- and intra-tumoral heterogeneity in both density and composition. Early immunohistochemical studies described dense peritumoral lymphocytic infiltrates composed mainly of CD3^+^ T-cells with variable CD4^+^/CD8^+^ ratios [15,16,17]. Terao et al. found that the CD4^+^ proportion was 19.1% ± 6.3% compared to 31.4% ± 3.5% CD8^+^ T-cells in a study of four patients [15]; while Haeffner et al. used one cSCC sample, which was for IHC, which showed 43% CD4^+^ T-cells and 26% CD8^+^ T-cells. CD3^+^ T-cells were the main constituent of cells at 75.2% [17]. Modern work capitalized on high-resolution spatial and single-cell analyses to uncover distinct microanatomical niches reflecting immune activation, exhaustion, or exclusion [18,19,20]. In particular, exhaustion scores (calculated according to RNA sequencing signals) were markedly increased in recurrent cSCC compared to primary cSCC, and activity scores for TNF and NOD-like receptor signaling pathways showed significantly decreased levels in recurrent cSCC.

Overall, TILs largely consist of CD3^+^ T-cells, which classically include CD4^+^ and CD8^+^ T-cells. These cells are often more abundant in higher-stage or poorly differentiated lesions [19,21,22,23]. In comparison to non-cancerous lesions, cSCC had significantly increased amounts of activated CD8^+^ T-cells (30.1% in cSCC, 18.2% in keratoacanthoma in a study of 13 cSCC) [19]. Satchell et al. found SCC was more likely to be infiltrated by CD3^+^ T-cells (20 of 20 samples, median 55 cells/mm^2^) compared to actinic keratosis (13 of 19 samples, median 20 cells/mm^2^) [24]. TILs increased significantly and in a stepwise fashion from normal skin to pre-cancerous lesions and subsequently cancer. This is revealed by histological scores on a study of 125 patients with cSCC (Klintrup–Mäkinen (KM) Score 1.8, standard deviation (SD) 0.85) compared to adjacent normal skin (KM score 0.82, (SD) 0.46), Actinic Keratosis (AK) (KM score 1.28, SD 0.52) and SCC in situ (KM score 1.66, SD 0.63) [25], building on Stravodimou et al.’s previous results where CD8^+^ T-cells showed the same pattern but it was noted that aggressive types of SCC (acantholytic/adenoid, adenosquamous, spindle cell and desmoplastic subtypes) had fewer CD3^+^ cells [21]. Bauer et al. also showed increased CD3^+^ and CD8^+^ cells in cSCC in a study comparing 106 samples of cSCC to AK [22].

Quantitative analyses using digital pathology showed a significant positive correlation between high VISTA expression and intratumoral CD3^+^, CD4^+^Treg, and CD8^+^ infiltration [26], while PD-L1 expression in tumor cells correlated specifically with CD3^+^ and CD8^+^ infiltration within tumor nests [26,27,28]. No significant correlation was reported between tumor grade and CD3^+^ density [26]. Both cytotoxic-exhausted CD8^+^ T-cells (CTLA4^+^) and FOXP3^+^ TIGIT^+^ Treg cells increased progressively from normal skin through precursor lesions to cSCC [29].

CD3^+^ TILs accumulated in the peritumoral rather than intratumoral space, and also gradually increased when closer to cSCC tissue in two studies with a combined 151 samples [30,31]. CD8^+^ T-cells also showed the same pattern, with one study of 24 samples indicating all entities showed more cytotoxic T-cells in the peritumoral area rather than intra-tumorally [32]. CD4^+^ helper T-cells were frequently present within peritumoral stroma and can comprise 50–60% of total T-cells in cSCC [33], but were often outnumbered by cytotoxic CD8^+^ subsets in high-grade lesions [30,31,34]. CD8^+^ cytotoxic T-cells typically infiltrated tumor nests and stroma and showed variable proliferation, activation, and apoptotic markers [35,36,37]. High intratumoral CD8^+^ density correlated with PD-L1 expression [27,31,38], VISTA positivity, and upregulated cytotoxic markers such as granzyme B (GzmB) and Ki-67 [26]. Conversely, organ-transplant recipients and immunosuppressed individuals displayed markedly fewer CD8^+^ and granzyme B^+^ cells [39,40].

Other CD3^+^ TILs that have been characterized include CD103^+^ tissue-resident memory T-cells (TRM), which were associated with durable local immune surveillance. However, in cSCC, these cells exhibited reduced protective activity and lower effector cytokine production compared with healthy skin [41].

A subset of these CD4^+^ T-cells co-expressed FOXP3, indicating the presence of regulatory T-cells (Treg), which Lai et al. quantified at 29.2 ± 19.4% of T-cells in a sample of 79 cSCC [30]. Treg cells were more numerous in cSCC (19.8% ± 8.6%) compared to normal skin (7.3% ± 4.1%), were markedly enriched in invasive or metastatic tumors (49.3% ± 13.8% in cSCC that metastasized compared to 23.5% ± 11.0% in cSCC that did not metastasize) and correlated with reduced CD8^+^ infiltration and function (median suppression 41.7%) and poorer differentiation [30,33,42]. Tregs were also consistently elevated in cSCC relative to precursor lesions such as actinic keratosis or Bowen’s disease [21,24,42,43], as well as mimics like keratoacanthoma (33.7% in cSCC compared to 22% in KA in a study comparing 13 samples each of cSCC and keratoacanthoma) [19], suggesting progressive immunosuppression during tumorigenesis. OX40^+^FOXP3^+^ Tregs demonstrated strong suppressive function (where addition of anti-OX40 improved T cell proliferation by 252.4%) and was associated with metastatic potential and reduced effector T-cell activation [30].

Regarding the TIL function, studies identified robust expression of activation and memory markers, which signifies active antigen presentation in the tumoral microenvironment. CD45RO^+^ memory T-cells predominated over CD45RA^+^ naïve cells in most samples where investigated [21,34,41]. CD69^+^ (an activation marker) was enriched in invasive tumors, while CCR7 and CD27/CD28 naïve phenotypes were scarce, consistent with an effector-memory skew.

Although an effector memory response may potentially enhance anti-tumoral immunity, studies have also revealed frequent expression of exhaustion markers amongst TILs. These markers include CD39, Tim-3, and LAG-3, and they were frequently expressed in TILs of advanced or recurrent cSCC [44,45,46]. For example, LAG-3 negative tumors had a mean diameter of 3.5 cm compared with LAG3-positive tumors, which had a mean diameter of 8.5 cm in 1 study that studied cSCC from 13 patients [44]. LAG-3 (found in 50.8% of CD8^+^ lymphocytes, compared to 35.2% of PD-L1-positive CD8^+^ lymphocytes in a study with samples from 3 patients) may be a useful marker of advanced cSCC [46]. CD39 expression also correlated with UVR-induced DNA damage, impaired repair capacity, and metastasis [45].

Another facet of anti-tumoral immunity pertains to immune checkpoints, which have been well-documented to be exploited by cancers for immune evasion. Indeed, PD-L1 expression in tumor cells increased with disease progression, nodal metastasis, and poor differentiation [25,26,27,28,31,38,47,48,49,50]. Allred scores for both epithelial PD-L1 (ePD-L1) and stromal PD-L1 (sPD-L1) expression were highest in invasive squamous cell carcinoma (inSCC), with mean ePD-L1 of 1.2 (SD 1.8) and sPD-L1 of 3.0 (SD 1.84), compared with progressively lower expression in in situ SCC (isSCC; ePD-L1 0.44, SD 1.24; sPD-L1 1.69, SD 1.3), actinic keratosis (AK; ePD-L1 0.1, SD 0.52; sPD-L1 1.12, SD 1.43), and normal skin (NS; ePD-L1 0; sPD-L1 0.36, SD 0.94). Both ePD-L1 and sPD-L1 expression demonstrated positive correlations with inSCC tumor thickness. In addition, ePD-L1 expression correlated positively with tumor diameter as well as with stromal and epithelial CD3^+^ and CD8^+^ tumor-infiltrating lymphocytes [25]. Correspondingly, PD-1^+^ TILs co-localized with exhausted CD8^+^ subsets, signifying suppression of cytotoxic T-cell activity in the tumor microenvironment. High Coatomer Protein Complex Subunit Beta 2 (COPB2) expression in cSCC was associated with increased densities of both CD4^+^ and CD8^+^ TILs, yet paradoxically, these COPB2-high tumors demonstrated worse recurrence-free survival [51]. Intratumoral and peritumoral CD4^+^ and CD8^+^ TILs were significantly increased in cSCC (Intratumoral CD4^+^ 59.1%, peritumoral CD4^+^ 68.2%, intratumoral CD8^+^ 63.6%, peritumoral CD8^+^ 72.7%) with high COPB2 compared to those with low COPB2 (Intratumoral CD4^+^ 19.2%, peritumoral CD4^+^ 39.7%, intratumoral CD8^+^ 27.4%, peritumoral CD8^+^ 37.0%).

These findings are summarized in Table 1 and highlight that the cSCC immune microenvironment transitions from an initially durable anti-tumoral response dominated by cytotoxic and memory T-cells to a suppressive milieu characterized by immune checkpoint activation, pathological Treg activity, and T-cell exhaustion.

### 3.2. Comparison of Tumor-Infiltrating Lymphocytes Between Immunocompetent and Immunosuppressed Cutaneous Squamous Cell Carcinoma

The immune composition of cutaneous squamous cell carcinoma (cSCC) differs markedly according to host immune status. Nineteen of the included studies specifically contrasted immunocompetent with immunosuppressed settings, encompassing solid-organ transplant recipients, patients with epidermolysis bullosa (EB), and individuals on long-term immunomodulators [18,20,22,25,26,31,34,38,39,40,42,45,52,53,54,55,56,57,58].

#### 3.2.1. Overall TIL Density

Studies reported a substantial reduction in total CD3^+^ T-cell density in immunosuppressed patients. Strobel et al. quantified a >50% decrease in total TIL counts and demonstrated altered spatial distribution—T-cells in transplant-associated cSCC accumulated peripherally rather than infiltrating tumor nests from their study comprising 20 immunocompetent and 20 organ transplant recipients with cSCC [39]. The overall density of tumor-infiltrating lymphocytes (TILs) was lower in organ transplant recipients (OTRs) than in immunocompetent individuals, with a median of 401 cells/mm^2^ (range 0–7976) in the OTR group compared with 940 cells/mm^2^ (range 0–12,507) in the immunocompetent group. In both populations, the intratumoral compartment harbored the lowest density of infiltrating leukocytes, measuring a median of 510 cells/mm^2^ (range 0–10,943) in immunocompetent patients and only 90 cells/mm^2^ (range 0–7900) in OTRs, when contrasted with surrounding non-tumoral tissue. By contrast, immune infiltrates at the directly tumor-adjacent invasive margin (IM500: 0–500 um from tumor edge) were consistently denser than intratumoral infiltrates in both groups, with medians of 1940 cells/mm^2^ (range 0–11,992) in immunocompetent patients and 680 cells/mm^2^ (range 5–7976) in OTRs. Notably, immune cell density at the invasive margin was significantly reduced in SCCs arising in OTRs compared with immunocompetent individuals. Spatial analysis further demonstrated that CD4^+^ T-cell infiltration was significantly diminished in OTRs at both defined peritumoral zones: at IM1500 (500–1500 um from tumour edge), median CD4^+^ density was 1512 cells/mm^2^ (range 0–4475) in OTRs versus 2651 cells/mm^2^ (range 0–12,507) in immunocompetent patients, while at IM500, CD4^+^ densities were likewise lower in OTRs (1706 cells/mm^2^, range 448–7976) compared with immunocompetent individuals (4180 cells/mm^2^, range 0–11,992). Similarly, Frazzette et al. observed a twofold reduction in cytotoxic T-cells (6880 cells in immunocompetent patients compared to 2484 in immunocompromised patients, by flow cytometry) and markedly decreased TCR clonality (average of 1140 clonotypes in immunocompetent patients compared to an average of 544 clonotypes in immunocompromised patients) [40], suggesting a restricted TIL repertoire under chronic immunosuppression. A reduced number of inflammatory infiltrate was also observed in EB-associated SCC, with significantly lower numbers of CD4^+^ T-cells (mean: 649 cells/mm^2^) and CD8^+^ T-cells (mean 794 cells/mm^2^) compared with SCCs from immunocompetent patients (mean CD4^+^: 1211 cells/mm^2^, mean CD8^+^ T-cells: 1228/mm^2^) [58].

#### 3.2.2. Subtype Composition

The CD8^+^ cytotoxic compartment was disproportionately diminished compared with CD4^+^ helper subsets, resulting in an inverted CD4:CD8 ratio in many transplant-associated cases [34,39,40]. Feldmeyer et al. quantified the upregulation of FOXP3 expression in 50 organ transplant recipient samples (mean 5.6×), as well as surface molecules and transcription factors associated with Tregs including IL2R, TGFb1 (mean 2.5×), ID3 (mean 5.3×), RUNX3 (mean 2.16×), FOXO1 (mean 1.9×), OX40 (mean 2.2×) and CXCR4 (mean 3.5×). Bottomley et al. also showed CD8^+^ T-cells were dramatically reduced in cSCC from kidney transplant recipients (10–40 peritumoral, 1–10 intratumoral) compared to immunocompetent (70–620 peritumoral, 12–280 intratumoral) [59]. In addition to reduced abundance, residual CD8^+^ cells often showed higher expression of exhaustion markers such as PD-1, LAG-3, and TIM-3 [44,46,58], which entail a suboptimal cytotoxic response in the tumor microenvironment. In contrast, FOXP3^+^ regulatory T-cells and OX40^+^ Tregs were relatively enriched [30,33,42,58], driving an immunosuppressive milieu.

EB–associated SCCs shared a similar immunosuppressive signature, with elevated PD-L1 expression, increased FOXP3^+^ Tregs, and diminished effector infiltration [58]. PD-L1 expression was significantly higher in DEB-SCCs (mean H-score: 151.8), KEB-SCCs (mean H-score: 132.1), and IS-SCCs (mean H-score: 118.6) compared with IC-SCCs (mean H-score: 85.34).

#### 3.2.3. Checkpoint Expression and Activation

Checkpoint molecule expression was accentuated under systemic immunosuppression. PD-1 positivity was observed in up to 80% of transplant-related cSCC [31,38,53]. Varki et al. evaluated 66 patients and found an average of 83% of CD4^+^ T-cells that expressed PD-1, while an average of 88% of CD8^+^ T-cells had PD-1 [31]. This is compared with 55% in immunocompetent patients [53]. Conversely, markers of activation (CD69^+^, Ki-67^+^, GzmB^+^) were sparse, indicating significant checkpoint-mediated functional suppression of anti-tumoral immunity in such settings.

#### 3.2.4. Spatial and Functional Observations

Digital spatial profiling and single-cell data highlighted that immunosuppressed cSCCs lacked central T-cell infiltration and displayed stromal segregation of CD8^+^ cells, consistent with an “immune-excluded” phenotype [18,20,26]. Organ transplant-derived lesions further exhibited decreased Dendritic Cell (DC) and CD11c^+^ populations [39], weakening antigen presentation.

Collectively, immunosuppressed cSCCs are characterized by reduced TIL abundance and cytotoxic function. This is further compounded by ineffective localization of the anti-tumoral response in the tumor microenvironment (Table 2). 

### 3.3. External Modulators of the Tumor-Infiltrating Lymphocyte Landscape in Cutaneous Squamous Cell Carcinoma

Multiple therapeutic and microenvironmental modulators alter TIL composition and activation in cSCC. Six studies [30,32,45,56,60,61] experimentally or clinically investigated immunomodulatory interventions—including topical immune stimulants, vaccines, and combination regimens—while others provided mechanistic insight into tumor–immune crosstalk [45,59]. These results are summarized in Table 3. Separately, arsenic as an environmental toxin was associated with apoptosis of CD4^+^ T-cells [62].

#### 3.3.1. Co-Stimulatory Checkpoint Targeting (OX40^+^ Tregs)

Lai et al. reported that OX40^+^ regulatory T-cells in cSCC are immunosuppressive and correlate with metastatic potential [30]. However, in vitro blockade of OX40 enhanced CD8^+^ T-cell cytotoxicity and Interferon-γ (IFN-γ) secretion, suggesting that selective OX40 modulation could restore local effector dominance. The findings support anti-OX40 therapy as a rational adjuvant strategy in immune-cold cSCC.

#### 3.3.2. In Situ HPV Vaccination

Zhang et al. found that HPV vaccination significantly augmented local immune infiltration in vaccinated versus unvaccinated patients’ primary cSCCs [56]. IHC revealed higher expression of CD8^+^, CD4^+^, CD69^+^, CD11c^+^, and CD163^+^ cells, indicating increased recruitment of both effector T-cells and myeloid antigen-presenting subsets. This supports the concept that systemic antiviral immunity can indirectly enhance anti-tumor TIL responses.

#### 3.3.3. Topical Immune Stimulation (Imiquimod)

Huang et al. demonstrated that topical imiquimod enhances TIL functionality in human cSCC by increasing IFN-γ production and effector activation markers (CD69, GzmB) among infiltrating T-cells [60]. It also modulates T-cell ratios: untreated SCCs are infiltrated by equal numbers of CD4^+^ and CD8^+^ T-cells, whereas tumors treated with imiquimod prior to excision contain >90% CD8^+^ cytotoxic T-cells. An older study by Clark et al. also showed that SCC tumors treated with imiquimod contained decreased percentages of Treg cells (from 41% to 31%, which resulted from a marked influx of non-Treg cells into the tumor, most of which were cytotoxic CD8^+^ T-cells). Imiquimod only slightly reduced the proliferation of FOXP3+ Treg cells but significantly reduced the function of Treg cells. Inactivation of Treg cells required at least 3 days of imiquimod treatment [33]. Imiquimod-treated lesions displayed intensified peritumoral CD8^+^ infiltration and upregulation of cytotoxic factors. T-cells from imiquimod-treated SCC produced markedly elevated levels of IFN-γ (IFN-γ was produced by 69% of CD4 T-cells (SD = 3.1, N = 3) and 79% of CD8 T-cells (SD= 7.4, N = 3) from treated tumors, compared with 8.2% of CD4 (SD = 1.4, N = 3) and 21% (SD = 13, N = 3) of CD8 T-cells). CD4 and CD8 cells produced more granzyme (from 20% of CD4^+^ T-cells to 50%, from 53% of CD8^+^ T-cells to 88%), perforin (from 7% of CD4+ T-cells to 37%, from 22% of CD8^+^ T-cells to 86%) [60].

#### 3.3.4. In Situ Vaccination and Immune Checkpoint Synergy

Huang et al. described a clinical case where autologous CD16^+^ DC injection combined with anti-PD-L1 and local radiotherapy led to marked CD8^+^ and CD4^+^ T-cell infiltration, proliferation (Ki-67^+^), and activation (CD69^+^), accompanied by tumor regression [61]. This synergistic triple regimen underscores how radiation-induced antigen release and PD-L1 blockade can reactivate suppressed cytotoxic TILs in situ. Additionally, Yost et al. described the clonal replacement of exhausted CD8^+^ T-cells from novel clonotypes, from pre- and post-treatment samples of 4 patients treated with anti-PD1. PD-1 blockade enabled new tumor-specific T-cells to enter and expand, though clonal exhaustion still occurs in the cSCC microenvironment [52]. However, Schenck et al. did not find a significant increase or decrease in CD8^+^ T-cells in a sample of five patients treated with nivolumab after disease progression on tacrolimus and prednisolone in organ transplant recipients (one patient had increased CD8^+^ T-cells on IHC score, which decreased in another patient) [57].

#### 3.3.5. Neutrophil Extracellular Traps (NETs) and TIL Exclusion

Moeller et al. highlighted an inverse association between neutrophil extracellular traps (NETs) and CD8^+^ TIL density, particularly in ulcerated or high-risk cSCC [32]. High NET burden correlated with reduced CD8^+^ infiltration and greater tumor aggressiveness, suggesting NETs contribute to an immunosuppressive microenvironment by physically impeding lymphocyte access or releasing inhibitory mediators.

#### 3.3.6. TGF-β2 and Stromal Immuno-Exclusion

Bottomley et al. identified TGF-β2 as a central stromal signal driving TIL exclusion in cSCC [59]. High TGF-β2 expression at the invasive front correlated inversely with CD8^+^ cell density and was linked to enrichment of fibrovascular and endothelial populations that form “immuno-exclusive” niches. This mechanism was observed irrespective of immune status, indicating that stromal TGF-β2 activity universally dampens cytotoxic infiltration.

These findings collectively indicate that external immune modulation can either invigorate or inhibit TIL responses in cSCC depending on the dominant pathway targeted. Pro-inflammatory interventions (imiquimod, the HPV vaccine, DC + anti-PD-L1 + radiotherapy) enhance recruitment, activation, and cytotoxicity of TILs. Microenvironmental suppressors (NETs, stromal TGF-β2) impede CD8^+^ infiltration and foster immune exclusion. Checkpoint-targeting combinations show the greatest potential to reverse exhaustion, bridging innate and adaptive immunity.

Together, these data emphasize that modulation of both stromal architecture and TIL checkpoint balance is essential to shift cSCC from an immuno-silent to an immuno-reactive phenotype and can help to direct future research.

## 4. Discussion

### 4.1. Overview of the cSCC Tumor Immune Landscape

The collective evidence from 48 studies spanning over three decades underscores the complex and spatially heterogeneous immune microenvironment of cutaneous squamous cell carcinoma (cSCC). The earliest studies that described T-cells in cSCC in 1977 enumerated them in a descriptive manner with light microscopy; technology then was inadequate to describe T-cells in further detail [63,64]. Building on promising work in melanoma [65], subsequent studies in the 1990s characterized cSCC as a T-cell-rich tumor, dominated by CD3^+^ and CD4^+^ lymphocytes, but provided little insight into their function [15,16,17]. Through the 2000s, work on apoptotic signaling and regulatory pathways revealed that tumor cells could actively subvert immunity through Fas–FasL interactions, recruitment of FOXP3^+^ regulatory T-cells, and downregulation of vascular adhesion molecules, establishing the concept of tumor-driven immune evasion [24,33,35,36].

From 2010 onward, checkpoint biology emerged as a central paradigm, with the identification of PD-1/PD-L1, OX40, and VISTA pathways, and comparative analyses between immunocompetent and immunosuppressed patients highlighting the profound influence of systemic immune status on local TIL composition [23,27,28,30,31,34,39,47]. In the past five years, single-cell sequencing, multiplex immunofluorescence, and spatial transcriptomics have revealed fine-grained immune zonation within tumors—cytotoxic T-cells concentrated at invasive margins, and regulatory or exhausted populations enriched within tumor cores [18,19,20,26,59].

Collectively, these advances delineate a progressive evolution from quantitative enumeration to functional and spatial mapping of immune cell subsets, reframing cSCC as a tumor in dynamic equilibrium between effector activation and immune tolerance. This framework provides the foundation for distinguishing the tumor-restraining and tumor-permissive lymphocyte populations that define cSCC’s immunologic phenotype. While cSCCs generally display prominent tumor-infiltrating lymphocytes (TILs), their functional balance between cytotoxic and immunosuppressive subsets determines tumor progression and therapeutic responsiveness. The cSCC immune microenvironment is not merely permissive but actively sculpted by tumor–immune crosstalk, generating zones of immune privilege that foster tumor persistence despite abundant lymphocytic presence.

### 4.2. Tumor-Restraining Immune Subsets and Mechanisms

Cytotoxic CD8^+^ T-cells constitute the principal effector population mediating tumor clearance. Multiple studies demonstrate that higher densities of CD8^+^ TILs correlate with improved local control (smaller tumor diameter, lower stage, and absence of metastasis) and disease-free survival [26,27,47,49,60]. These lymphocytes frequently co-express granzyme B (GzmB) and Ki-67, reflecting cytotoxic ability and proliferative activation, particularly in PD-L1-high tumors or following immune stimulation [30,60,61]. CD69^+^ and CD45RO^+^ memory T-cells further contribute to durable immunosurveillance [41,60]. However, the spatial context is critical: intratumoral and peritumoral densities may diverge, and tumors with fibrovascular or TGF-β–dominant stroma exhibit exclusion of CD8^+^ cells from the tumor parenchyma, thereby diminishing their functional impact.

CD103^+^ tissue-resident memory cells (TRMs) mark localized immunity at epithelial interfaces [41]. While they are enriched in the normal skin adjacent to cSCC, their proportion and functional activity are often reduced within tumor tissue, indicating impaired retention or exhaustion within the malignant niche. Increased CD103^+^ TRM density above 9.25% is associated with higher metastatic potential and recurrence risk. This pool of appropriately localized, potentially protective subsets represents a suitable target for hampering cSCC progression, especially in early disease. In melanoma, TRM cells has recently been shown in a murine model to promote a durable melanoma-immune equilibrium that is confined to the epidermal layer of the skin—TRM-deficient mice were more susceptible to tumor development, tumor-specific TRM cells generated prior to melanoma inoculation protected against tumor development, and TRM depletion promoted tumor outgrowth in 20% of mice with occult melanomas [66].

CD4^+^ helper T-cells, particularly T helper type 1 (Th1) polarized populations, augment tumor-restraining responses by producing IFN-γ and IL-2, and by supporting cytotoxic priming. Their abundance increases in HPV-vaccinated patients and after topical immune modulation with imiquimod. Similarly, DC recruitment (CD11c^+^) and macrophage co-activation (CD163^+^) accompany enhanced T-cell function in vaccinated or imiquimod-treated tumors, illustrating the synergy between innate and adaptive arms of cutaneous immunity [56,60].

Emerging checkpoint and co-stimulatory markers refine this understanding. Upregulation of PD-1^+^, TIM-3^+^, and LAG-3^+^ cells within active TIL populations often signifies adaptive resistance rather than exhaustion, as many retain proliferative and cytotoxic markers [26,44,46]. Inhibition of these checkpoints, particularly PD-1/PD-L1, restores effector dominance and yields clinical responses in advanced disease [53,57]. Similarly, OX40 modulation enhances IFN-γ production, supporting co-stimulatory checkpoint agonists as viable therapeutic adjuvants [30].

Collectively, these data identify a subset of tumor-restraining, pro-immunogenic lymphocytes—CD8^+^GzmB^+^, CD4^+^IFN-γ^+^, CD103^+^ TRM, and CD69^+^ memory cells—whose activation predicts favorable outcomes and treatment responsiveness.

### 4.3. Tumor-Permissive Immune Subsets and Immunosuppressive Mechanisms

Opposing these beneficial cells are multiple tumor-permissive immune elements that mediate local tolerance and immune evasion. Chief among them are FOXP3^+^ regulatory T-cells (Tregs), which are enriched in poorly differentiated, metastatic, or transplant-related cSCC [33,39,42], exerting suppression through cytokine production and checkpoint signaling. Their infiltration is accompanied by increased expression of CCR4, OX40, and PD-1, reflecting activated yet suppressive phenotypes [30,31]. In organ transplant recipients and immunosuppressed patients, CD8^+^ density and clonality are reduced while Treg frequency doubles, reinforcing a microenvironmental shift toward immunosuppression [34,39,40].

Stromal and cytokine-driven pathways further exacerbate immune exclusion. Overexpression of TGF-β2 at the tumor–stroma interface correlates inversely with CD8^+^ density and promotes mesenchymal remodeling, resulting in “immuno-exclusive” zones devoid of effector cells [59]. This is accompanied by PD-1, TIM-3, LAG-3, and CD39 expression in advanced cSCC, suggesting that stromal and regulatory signaling act synergistically to suppress cytotoxic T-cell infiltration and effector function. In this context, T-cell exhaustion represents a distinct, tumor-permissive differentiation state arising from chronic antigen exposure within the cSCC microenvironment. Exhausted CD8^+^ T-cells progressively lose effector functions, including proliferative capacity, cytokine production, and durable cytotoxicity, while failing to persist as long-lived memory cells. This state is characterized by coordinated upregulation of inhibitory receptors such as CTLA-4, PD-1, LAG-3, TIM-3, and TIGIT, which transduce suppressive signals that restrain T-cell activation despite ongoing antigen recognition [67]. Importantly, exhaustion in cSCC does not imply immune absence; it reflects sustained immune engagement that is functionally constrained by checkpoint signaling, stromal exclusion, and regulatory cytokines. This paradigm explains the frequent observation of dense yet ineffective T-cell infiltrates in advanced cSCC and provides a mechanistic basis for the clinical efficacy of immune checkpoint blockade, which partially reinvigorates exhausted but tumor-reactive T-cell populations [19,21,22,23,24,25,30,31,44,45,46,51].

Similarly, neutrophil extracellular traps (NETs) correlate inversely with CD8^+^ infiltration and are associated with ulceration and advanced stage, suggesting that neutrophils act as physical and biochemical barriers to lymphocyte access [32].

At the tumor cell level, PD-L1, VISTA, COPB2, and FOXP3 expression on malignant keratinocytes themselves contribute to bidirectional immunosuppression [26,31,51]. A recent study on pre-treatment and post-treatment biopsies from 12 patients who received cemiplimab (an anti-PD1 therapy) showed that responder patients displayed decreased T helper and CD4^+^ T-cells in tumor specimens after anti-PD1 therapy, along with downregulation of Tumor Necrosis Factor signaling, IL-1 β, and IL-8, whereas CD8^+^ T-cells and B cells increased. Non-responder tissues showed the opposite trend [68]. High VISTA and COPB2 correlate with both increased T-cell infiltration and Treg enrichment, indicating a complex compensatory mechanism balancing activation and tolerance. Yet, COPB2 has been shown to correlate with poorer recurrence-free survival, supporting the concept that some cSCCs display an “inflamed yet ineffective” immune phenotype in which effector cells are present but functionally neutralized. These layers of immune regulation establish cSCC as a tumor with robust infiltration but incomplete immune effectiveness.

### 4.4. Integrating Immune Competence and Microenvironmental Context

Comparison across immunocompetent and immunosuppressed populations reveals that systemic immune status profoundly dictates local TIL composition. Immunosuppressed patients exhibit decreased CD8^+^ and TCR clonality, alongside expansion of FOXP3^+^ and PD-1^+^ exhausted T-cells [34,39,40]. The relative abundance of exhausted or anergic T-cells increases, while proliferative indices (Ki-67^+^) decline. While absolute TIL numbers are reduced, intra- and intertumoral heterogeneity remains preserved, implying that the suppressive TME is driven by the quality rather than quantity of TILs. Conversely, immunocompetent individuals maintain higher proportions of proliferating cytotoxic subsets expressing Ki-67, GzmB, and PD-1, suggesting dynamic but functional immune surveillance. Comparative studies between immunocompetent and immunosuppressed individuals reveal that fibroblastic and endothelial networks in transplant-associated cSCCs form physical and cytokine-mediated barriers that exclude effector lymphocytes, a phenomenon mirrored by elevated TGF-β2 and VEGF expression. These contrasts support the paradigm that effective anti-tumor immunity requires both competent systemic priming and permissive local architecture.

**Figure 2:**
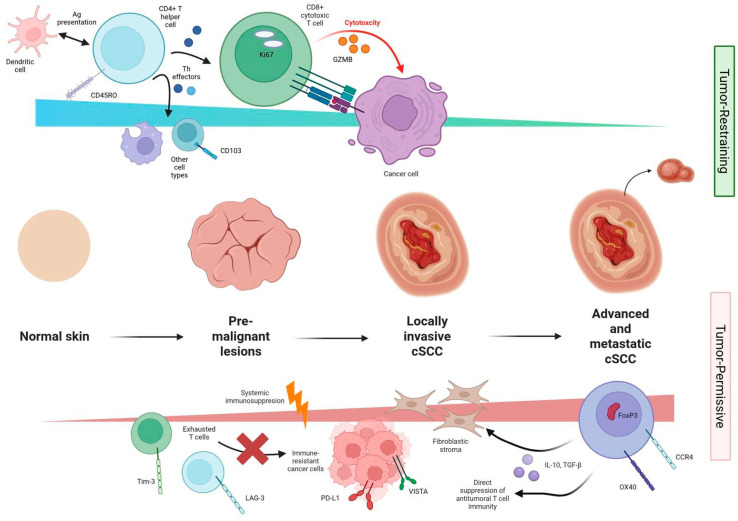
Immunogenic duality in cSCC progression (created in Biorender).

The reciprocal axes of immunity define cSCC’s “immunological equilibrium”, a dynamic balance between local cytotoxicity and tolerance, tabulated in Table 4 and graphically depicted in Figure 2. Therapeutic modulation should therefore aim not only to amplify effector arms but also to dismantle structural and cellular barriers that preclude their action.

### 4.5. Future Directions

Several key priorities emerge from the synthesis of current evidence on cSCC TIL biology. First, standardization of TIL assessment—including markers, counting methods, and spatial localization—remains essential to enable reproducibility across studies. Second, the functional and spatial characterization of TIL subtypes, particularly CD103^+^ tissue-resident memory cells and CD39^+^ exhausted phenotypes, warrants deeper investigation using multiplex spatial and single-cell platforms, leveraging multiplex imaging and single-cell transcriptomics to delineate the precise topography of TIL subsets. Third, longitudinal profiling of cSCC lesions, including precursor lesions like actinic keratosis, Bowen’s disease, and subsequently invasive or metastatic cSCC, may clarify when and how immune escape occurs. Fourth, transplant recipients and immunosuppressed cohorts must be prospectively studied under carefully controlled conditions to evaluate the safety and efficacy of immunotherapy, moving beyond exclusion toward personalized immune modulation.

Appreciating TIL biology in non-cSCC squamous cell carcinomas also offers meaningful perspectives that will guide future work. In cervical SCC, the CD8^+^/Treg ratio is a positive prognostic indicator [69,70], which is immunologically consistent with cSCC pathophysiology. However, reduced CD4^+^ T-cell levels appear to be associated with advanced disease and recurrence [71], which may be influenced by the complex interplay between anti-tumoral and anti- alpha-human papillomavirus (α-HPV) immunity. The prominence of α-HPV in cervical SCC as a proven oncovirus also offers druggable targets for immunotherapy and vaccination—a circumstance that is still nascent in cSCC, although there is growing evidence for the role of beta-human papillomavirus in carcinogenesis [72,73]. In head and neck SCC (hnSCC), dense CD3^+^ and CD8^+^ infiltration is similarly associated with prolonged overall survival, progression-free survival, and distant metastasis-free survival [74]. Overall, bolstering CD8^+^ TIL-driven anti-tumoral immunity is likely to be an important cog in the armamentarium against cSCC and its disease counterparts.

While there are studies that detail the characteristics of TILs in influencing the tumor response to immune checkpoint blockade in melanoma and other solid tumors [75,76], such studies are not yet available for cSCC and may merit further work to better prognosticate and determine other treatment pathways and adjuncts to immune checkpoint blockade. Therapeutically, combining checkpoint blockade (PD-1, VISTA, LAG-3) with co-stimulatory agonists (OX40) and microenvironment-targeting agents (anti-TGF-β, NET inhibitors) holds promise for converting immune-excluded tumors into inflamed, responsive phenotypes. HPV vaccination may play a role in reducing cancer incidence and progression for at-risk populations. Eventually, personalized approaches that integrate immunogenomic profiling with functional readouts of TIL exhaustion or activation could guide treatment selection, particularly in immunosuppressed or transplant-dependent patients. Ultimately, advancing from descriptive to mechanistic immuno-pathology will be key to transforming cSCC from a model of immune resistance into one of immunotherapeutic success.

## 5. Conclusions

This systematic review synthesizes three decades of human data to demonstrate that the immune microenvironment of cutaneous squamous cell carcinoma is defined not by the mere presence of tumor-infiltrating lymphocytes, but by the balance between tumor-restraining cytotoxic immunity and tumor-permissive immunosuppressive mechanisms. cSCCs commonly harbor dense CD3^+^ infiltrates, yet effective tumor control is associated with spatially accessible, proliferative CD8^+^ effector and tissue-resident memory populations, whereas disease progression correlates with regulatory T-cell enrichment, stromal remodeling, and cytokine-driven immune exclusion. Immune competence profoundly shapes these dynamics, with immunosuppressed states characterized by reduced cytotoxic clonality and expansion of suppressive networks despite preserved heterogeneity. External immune modulation can recalibrate this equilibrium, highlighting the therapeutic potential of combining checkpoint inhibition with strategies that enhance antigen presentation or dismantle stromal barriers. Collectively, these findings position cSCC as a model of immune engagement constrained by local tolerance, underscoring the need for spatially informed, context-specific immunotherapeutic approaches and standardized immune profiling to improve consolidation of data, prognostication, and guide future clinical translation in dermatology and dermatopathology.

## Figures and Tables

**Table 1:** Characteristics of Tumor-Infiltrating Lymphocytes in Cutaneous Squamous Cell Carcinoma.

TIL Subtype	Findings in cSCC	Associated Features	References
CD3^+^ T-cells	Major T-cell subset in cSCC Higher density in higher-stage and poorly differentiated lesions	Increased infiltration with VISTA expression and PD-L1-high tumors No correlation with tumour grade	[15,16,17,21,22,26,27,34,39,40]
CD4^+^ helper T-cells	Present mainly in peritumoral stroma Variable proportion compared to CD8^+^	Reduced in advanced or metastatic disease Associated with immunosuppressive phenotype and Treg differentiation COPB2 expression is associated with increased density but poorer recurrence-free survival Spatially graded increase in TIGIT^+^ Treg cells towards cSCC tissue	[29,30,31,34,51]
CD8^+^ cytotoxic T-cells	Intratumoral infiltration Key effector subset	Increased in VISTA-high and PD-L1-high tumors; reduced in immunosuppressed hosts Correlates with granzyme B and Ki-67; inversely related to FOXP3^+^ Tregs COPB2 expression is associated with increased density but poorer recurrence-free survival Progressive increase in CD8^+^ exhausted T-cells expressing high CTLA4 as tissue transitions from normal → AK → cSCC	[26,27,29,30,31,33,38,39,40,42,51]
CD103^+^ TRM cells	Resident memory T-cells with limited effector function	Decreased cytotoxicity and cytokine secretion in cSCC Impaired protective immunity in tumor-bearing skin	[41]
FOXP3^+^ Tregs	Abundant in invasive/metastatic tumors	FOXP3^+^ density is higher in invasive vs. in situ disease Suppresses effector T-cell activity Correlates with OX40^+^ expression and metastasis	[21,30,33,42,43]
CD45RO^+^ memory T-cells	Dominant memory phenotype	Predominate over CD45RA^+^ naïve subsets Indicates prior activation; higher in immunocompetent hosts	[21,34,41]
CD69^+^ activated T-cells	Present in invasive tumors	Increased relative to precursors Activation marker upregulated in high-risk cSCC	[21,41]
CD45RA^+^/CCR7^+^ naïve T-cells	Low in the tumor microenvironment	Indicates effector-memory shift	[21,34,41]
CD27/CD28 subsets	EM1–EM4 heterogeneity in effector memory cells	Skewed toward EM2/EM3 in invasive disease Reflects chronic antigen exposure	[41]
CD39^+^ exhausted T-cells	Expressed in metastasizing cSCC	Associated with UVR-induced DNA damage and metastasis Exhaustion marker; decreased repair capacity	[45]
Tim-3^+^/LAG-3^+^ T-cells	Frequently expressed in advanced or recurrent tumors	Qualitatively higher in late-stage disease Co-expression with PD-1 indicates exhaustion	[44,46]
PD-L1 (tumor)	Upregulated with progression	Increased in metastatic and poorly differentiated tumors. Correlates with CD3^+^, CD8^+^ infiltration and T-cell activation	[25,26,27,28,31,38,47,48,49,50]
PD-L1 (lymphocytes)	Found in exhausted cytotoxic T-cells	Increased with PD-L1 expression Reflects immune checkpoint engagement	[26,31,44,46]
Ki-67^+^ proliferating T-cells	Present in VISTA- and PD-L1-high tumors	Ki-67^+^CD3^+^ proliferation enriched in those groups Indicates activated cytotoxic state	[26]
GzmB^+^ cytotoxic T-cells	Parallel increase with Ki-67^+^	GzmB^+^CD3^+^ enriched in VISTA/PD-L1-high cSCC Reflects active killing potential	[26]

**Table 2:** Differential TIL and checkpoint characteristics in immunocompetent vs. immunosuppressed Cutaneous squamous cell carcinoma.

TIL Subtype	Immunocompetent	Immunosuppressed	Differences	References
Total CD3^+^ TIL density	Dense intratumoral and stromal infiltration	Sparse, peritumoral localization	↓ >50% density; altered spatial pattern	[34,39,40,58]
CD4^+^ helper T-cells	Moderate numbers; assist effector activation	Variable; may predominate over CD8^+^	CD4:CD8 ratio ↑ in transplant patients	[34,39,40]
CD8^+^ cytotoxic T-cells	Abundant; intratumoral localization	Markedly reduced; peripheral clustering	2–3× lower density; decreased TCR clonality	[39,40,58]
CD103^+^ TRM	Present; supports local surveillance	Rare; reduced residency markers	Loss of tissue-resident phenotype in immunosuppression	[18,20,41]
FOXP3^+^ Tregs	Present but balanced with cytotoxic cells	Enriched; peritumoral accumulation	↑ FOXP3^+^ frequency; FOXP3:CD8 ratio > 1 in EB-SCC	[30,33,39,42,58]
CD45RO^+^ memory T-cells	Predominant memory phenotype	Reduced the memory compartment	↓ memory/naïve ratio	[34,40]
Activation markers (CD69^+^, Ki-67^+^, GzmB^+^)	Frequent in active TILs	Rare or absent	↓ proliferating and cytotoxic fractions	[26,40]
Exhaustion markers (PD-1, CD39, Tim-3, LAG-3)	Moderate expression, reversible	Strong, sustained expression	↑ exhaustion marker co-expression	[44,45,46,58]
PD-L1 expression (tumor)	40–60% positive; linked to high TIL density	70–80% positive; not linked to TIL density	↑ expression, but functionally non-productive	[27,28,31,38,49,53,58]
DC/CD11c^+^ cells	Preserved antigen presentation	Reduced APC numbers	↓ DCs in transplant cSCC	[39]
Spatial architecture	“Inflamed” or “immune-hot”	“Immune-excluded” with stromal trapping	Central exclusion of CD8^+^ and CD4^+^ cells	[18,20,26,39]

Up arrow: Increased, Down arrow: Decreased.

**Table 3:** External modulators of tumor-infiltrating lymphocytes in Cutaneous squamous cell carcinoma.

Modulators	Mechanism	Observed Effect on TILs	References
OX40^+^ Treg modulation	Co-stimulatory checkpoint reversal	OX40 blockade → ↑ IFN-γ secretion, ↑ cytotoxicity, ↓ suppressive Tregs (ex vivo)	[30]
Anti PD-1 therapy	Enables new tumor-specific T-cells to enter and expand	Clonal replacement of exhausted CD8^+^ T-cells from novel clonotypes	[52]
HPV vaccination	Vaccine-driven immune priming	↑ CD8^+^, CD4^+^, CD69^+^, CD11c^+^, CD163^+^ infiltration in vaccinated patients	[56]
Imiquimod (topical)	TLR7 agonist → ↑ IFN-γ, granzyme, perforin	↑ CD8^+^, ↑ GzmB^+^ TILs; enhanced effector activation	[60]
Triple regimen (autologous CD16^+^ DC + anti-PD-L1 + radiotherapy)	Antigen release + checkpoint blockade synergy	↑ CD8^+^, CD4^+^, Ki-67^+^, CD69^+^ infiltration; enhanced tumor regression	[61]
Neutrophil extracellular traps (NETs)	Neutrophil-mediated immune barrier	High NET density → ↓ CD8^+^ infiltration, ↑ ulceration risk	[32]
TGF-β2 signaling	Fibrovascular stromal exclusion; immune suppression	↑ TGF-β2 correlated with ↓ CD8^+^, ↑ fibroblast/endothelial niche formation	[59]

Up arrow: Increased, Down arrow: Decreased.

**Table 4:** Tumor-restraining and tumour-permissive environment.

Tumor-Restraining Elements	Tumor-Permissive Elements
CD8^+^ cytotoxic T-cells (GzmB^+^, Ki-67^+^)	FOXP3^+^ regulatory T-cells (CCR4^+^, OX40^+^)
CD4^+^ helper T-cells (IFN-γ^+^, IL-2^+^)	TGF-β2-driven fibroblastic stroma
CD69^+^ activated T-cells	PD-L1^+^, VISTA^+^, COPB2-high tumor cells
CD103^+^ tissue-resident memory T-cells	Neutrophil extracellular traps (NETs)
PD-1^+^/TIM-3^+^ exhausted yet recoverable T-cells	Organ-transplant–associated immunosuppression
CD45RO^+^ effector memory cells	Endogenous tumor FOXP3 expression

## Data Availability

No new data were created or analyzed in this study. Data sharing is not applicable to this article.

## Supplementary Materials

The following supporting information can be downloaded at: https://www.mdpi.com/article/10.3390/dermatopathology13010006/s1, PRISMA 2020 for Abstracts Checklist.

## Abbreviations

The following abbreviations are used in this manuscript:

cSCC	Cutaneous Squamous Cell Carcinoma
TIL	Tumor-Infiltrating Lymphocyte
PRISMA	Preferred Reporting Items for Systematic reviews and Meta-Analyses
CD	Cluster of Differentiation
CCR	C-C Chemokine Receptor
FOXP3	Forkhead box P3
TGF	Transforming Growth Factor
PD-1	Programmed Death
PD-L1	Programmed Death Ligand
HPV	Human Papillomavirus
OX40	Tumor Necrosis Factor Receptor Superfamily Member 4
TME	Tumor Micro-environment
Treg	Regulatory T-cells
LAG-3	Lymphocyte Activation Gene 3
VISTA	V-domain Immunoglobulin Suppressor of T-cell Activation
ICI	Immune Checkpoint Inhibitors
CTLA-4	Cytotoxic T lymphocyte-associated antigen 4
TCR	T-Cell Receptor
CAR	Chimeric Antigen Receptor
INPLASY	International Platform of Registered Systematic review and meta-analysis protocols
GzmB	Granzyme B
TRM	Tissue-Resident Memory T-cells
EM	Effector Memory
EB	Epidermolysis Bullosa
DC	Dendritic Cell
CXCL	C-X-C motif chemokine Ligand
NET	Neutrophil Extracellular Traps
IFN-γ	Interferon-γ
IHC	Immunohistochemistry
Th1	T helper type 1
COPB2	Coatomer Protein Complex Subunit Beta 2
